# Pushing the Limits of Interlimb Connectivity: Neuromodulation and Beyond

**DOI:** 10.3390/biomedicines13051228

**Published:** 2025-05-19

**Authors:** Jane A. Porter, Trevor S. Barss, Darren J. Mann, Zahra Karamzadeh, Deborah O. Okusanya, Sisuri G. Hemakumara, E. Paul Zehr, Taryn Klarner, Vivian K. Mushahwar

**Affiliations:** 1 Division of Physical Medicine and Rehabilitation, Department of Medicine, University of Alberta, Edmonton, AB T6G 2G3, Canada; jane.porter@queensu.ca (J.A.P.); djmann@ualberta.ca (D.J.M.); zkaramza@ualberta.ca (Z.K.); okusanya@ualberta.ca (D.O.O.); 2 Neuroscience and Mental Health Institute, University of Alberta, Edmonton, AB T6G 2R3, Canada; 3 Institute for Smart Augmentative and Restorative Technologies and Health Innovations (iSMART), University of Alberta, Edmonton, AB T6G 2R3, Canada; hemakuma@ualberta.ca; 4 Faculty of Kinesiology, Sport, and Recreation, University of Alberta, Edmonton, AB T6G 2S3, Canada; 5 Faculty of Rehabilitation Medicine, University of Alberta, Edmonton, AB T6G 2G4, Canada; 6 School of Exercise Science, Physical Education, and Health Education, University of Victoria, Victoria, BC V8W 2Y2, Canada; pzehr@uvic.ca; 7 International Collaboration on Repair Discoveries (ICORD), Vancouver, BC V5Z 1M9, Canada; 8 School of Kinesiology, Lakehead University, Thunder Bay, ON P7B 5E1, Canada; tklarner@lakeheadu.ca

**Keywords:** interlimb coordination, arm and leg cycling, rehabilitation, proprio-spinal connections, neuromodulation, transcutaneous spinal cord stimulation, functional electrical stimulation

## Abstract

The ability to walk is often lost after neural injury, leading to multiple secondary complications that reduce quality of life and increase healthcare costs. The current rehabilitation interventions primarily focus on restoring leg movements through intensive training on a treadmill or using robotic devices, but ignore engaging the arms. Several groups have recently shown that simultaneous arm and leg (A&L) cycling improves walking function and interlimb connectivity. These findings highlight the importance of neuronal pathways between the arm (cervical) and leg (lumbar) control regions in the spinal cord during locomotion, and emphasize the need for activating these pathways to improve walking after neural injury or disease. While the findings to date provide important evidence about actively including the arms in walking rehabilitation, these strategies have yet to be optimized. Moreover, improvements beyond A&L cycling alone may be possible with conjunctive targeted strategies to enhance spinal interlimb connectivity. The aim of this review is to highlight the current evidence for improvements in walking function and neural interlimb connectivity after neural injury or disease with cycling-based rehabilitation paradigms. Furthermore, strategies to enhance the outcomes of A&L cycling as a rehabilitation strategy are explored. These include the use of functional electrical stimulation-assisted cycling in acute care settings, utilizing non-invasive transcutaneous spinal cord stimulation to activate previously inaccessible circuitry in the spinal cord, and the use of paired arm and leg rehabilitation robotics. This review aims to consolidate the effects of exercise interventions that incorporate the arms on improved outcomes for walking, functional mobility, and neurological integrity, underscoring the importance of integrating the arms into the rehabilitation of walking after neurological conditions affecting sensorimotor function.

## 1. Introduction

Coordination between the arms and legs is a vital yet often overlooked component of walking that is important for the control of gait and balance in humans. The arms swing in counter-rotation to the legs while walking and running, which significantly increases the efficiency of gait, especially at faster speeds, and improves recovery from perturbations [1,2,3]. In both quadrupeds and humans, the rhythmic coordination between the arms and legs during locomotion results from neuronal networks between the cervical and lumbar enlargements in the spinal cord, as opposed to only interlimb mechanical linkages [2,4,5,6,7,8,9,10]. Long proprio-spinal neurons that connect these spinal cervicolumbar centers are most likely responsible for such coordination [5,6,11,12]. Evidence for interlimb coordination and reflex modulation in humans and animals [7,8,9] during locomotion demonstrates coordinated action by both arm and leg (A&L) control centers [4,6,10,13,14,15]. Similarly to quadrupedal mammals, a bidirectional linkage between the cervical and lumbar segments of the spinal cord during rhythmic movements is present in humans [4,11,12,15,16]. For example, leg H-reflexes are modulated during rhythmic arm movements and vice versa [12,16], and cutaneous reflexes are modulated between the upper and lower limbs during walking, but not during standing [17].

Neuronal networks do not fully regenerate after neural injuries; therefore, rehabilitation in the form of activity-based therapy has re-emerged as the most effective approach for restoring and improving function in the remaining pathways. The current prominent thinking in rehabilitation is that improvement in a task after neural damage can only be obtained by the repetitive training of that task. For example, for restoring walking after spinal cord injury (SCI) and stroke, body weight-supported treadmill training has been widely investigated as a task-specific intervention over the past 40 years [18,19,20,21,22,23]. Indeed, significant improvements in walking speed and distance were reported in people with incomplete SCI and stroke [24,25,26]. However, it has become apparent that engaging and strengthening the networks responsible for interlimb coordination through a simultaneous A&L cycling task can also significantly improve gait and balance after neural injury [27,28]. Specifically, legs-only cycling improves gait to similar levels seen with overground gait training and body weight-supported treadmill training [27]. Further, A&L cycling improves gait recovery by 2-fold more [27].

Several groups have studied how arm and/or leg cycling impacts the recovery of walking; however, to our knowledge, these results have not been examined collectively across intervention types and patient populations. The purpose of this narrative review is to summarize the existing findings from cycling exercise interventions in relation to improvements in walking and balance after neurological injury (SCI or stroke) or disease (multiple sclerosis, MS; or Parkinson’s disease, PD). Particular focus was placed on clinically important outcomes related to functional mobility in these clinical populations.

This review aims to synthesize clinical results from the published cycling interventions conducted in persons with neurological conditions to evaluate which components of these cycling interventions should be considered for future interventional optimization. We discuss the clinical performance of different cycling modalities (arm, leg, arm and leg, and FES inclusion) in each of the selected neurological conditions, concluding that interlimb connectivity is a crucial component of walking rehabilitation (Section 3). We also delve into modalities that show promise in harnessing interlimb connectivity to be explored in combination with A&L cycling for further optimization of intervention protocols (Section 4).

## 2. Methods

Information was gathered using a PubMed database search using keywords related to cycling or walking interventions, and clinical neurological conditions using common clinical assessments of locomotion. The search was conducted between fall 2021 and spring 2024 and multiple synonymous terms were used. Articles were included if the study group were persons with SCI, stroke, PD, or MS. Interventions were required to be longitudinal and include the desired clinical outcome measures in their analysis (Berg Balance scale, 10 m walk test, 6 min walk test). During the initial search period, articles with assessment using the Modified Ashworth Scale for spasticity were included; however, there were too few references with these measurements this metric was excluded from this review. Moreover, studies that did not include the populations and outcomes of interest were excluded. The gathered articles were evaluated to ensure the inclusion criteria were met, and decisions on inclusion were based on consensus.

The intervention characteristics are summarized in Table 1. Change in clinical test performance was collected from each article and evaluated by the authors. Change was reported both as absolute improvement and percent improvement, both of which were used to generate figures. Studies were segregated by study population in the creation of figures and discussion. Studies involving study participants with the same neurological condition were not placed into sub-groups based on condition severity due to the heterogeneity of the participants included within the available studies.

## 3. Cycling to Enhance Proprio-Spinal Connectivity and Improve Walking in Neurological Conditions Affecting Sensorimotor Function

Cycling provides a unique opportunity for activity-based therapy due to its inherently similar biomechanical properties and neural regulation to walking [47,48]. Previous investigations indicate background muscle activity and reflex amplitudes have similar phase dependency across walking, cycling, and stepping during rhythmic movement in humans. This is indicative of a common core regulating the pattern of activation [47]. Cycling allows individuals to participate in rehabilitation sooner after injury due to reduced dependence on balance control compared to walking [49]. Furthermore, the administration of cycling requires fewer therapists and less specialized equipment relative to alternatives, which improves cost and accessibility to rehabilitative care. The effectiveness of cycling as an exercise intervention for improving walking after neural injury or disease is demonstrated by its use across several clinical populations that include persons with SCI, stroke, PD, or MS [27,30,31,43].

This section summarizes the available evidence that cycling training improves walking. The included studies show outcomes from stationary cycling paradigms, which include legs-only cycling, arms-only cycling, and simultaneous A&L cycling. The selected studies are described alphabetically in Table 1. Each article was screened for the number of participants, clinical group, training mode, training frequency, and the type of cycling employed for training.

A particular focus was placed on clinically important outcomes related to functional mobility. These included the 10 min walk test (10MWT), 6 min walk test (6MWT), and the Berg Balance scale (BBS). Thus, the articles chosen for review included these measures before and after the investigated intervention. In addition to absolute and normalized scores, measurements of the minimal clinically important difference (MCID) and minimal detectable change (MDC) are reported. MCID is a score that refers to the minimum change that produces a noticeable difference by healthcare professionals or the individual themselves. MDC refers to the minimum amount of change that is considered a measurable difference (i.e., above the measurement error) for the population.

The 10MWT can be performed at a self-selected pace or at the fastest pace possible. Regardless of pace instruction, the participant is timed while they walk the middle 6 m of the 10 m total walking distance. For persons with incomplete SCI, the MCID on the 10MWT is 0.06 m/s [50]. However, the smallest difference in measurements beyond error, or the MDC, has been identified as 0.13 m/s [51]. These values are likely independent of the environment in which they are obtained [52]. For persons with stroke, the MCID is 0.16 m/s [53] and the MDC is dependent on the participant’s baseline gait speed and whether the 10MWT is conducted at a self-selected or fastest walking speed. In the fastest walking speed, changes of 0.04 m/s are detectable in low-speed walkers, whereas high-speed walkers require an improvement of 0.21 m/s for the change to be detectable [54]. Different reports exist on the MDC at self-selected and fast speeds for the 10MWT for persons with PD: the range at self-selected speed is 0.18–0.22 m/s and at fast speeds, the range is 0.23–0.25 m/s [55,56]. Persons with MS show an MDC of 0.26 m/s and an MCID between 0.08 and 0.19 m/s [57].

The 6MWT is a measurement of walking endurance where participants are given 6 min to ambulate as far as they can. Participants self-select their speed with breaks allowed throughout the test as long as they do not sit or lean against a support surface. The MCID for individuals with incomplete SCI is 36 m [58]. For persons with stroke, the MCID is 71 m in both the general sample and in people with gait speed above 0.4 m/s [59]. For persons with stroke who have baseline gait speeds below 0.4 m/s, the MCID is 44 m [59]. For people with PD, the MDC is 82 m [55]. A range of values for the MCID on the 6MWT is presented across the studies in persons with MS, with various participant cohorts: the values fall in the range of 21.56–55.06 m [57,60].

The BBS is a 56-point assessment of balance with 14 tasks, each scored in the range of 0–4. MDC or MCID values for SCI, specifically on the BBS, within the literature search conducted for this paper could not be found. The reported MDC values for persons with acute stroke range from 6.0 to 8.1 points on the BBS [61]. The MDC for persons with chronic stroke ranges from 2.7 to 4.7 points across studies [62,63]. For persons with PD, the MDC is a five-point increase [55], and the MCID for persons with MS is three points [64]. Assessing similar outcome measures across clinical conditions, exercise intensity, duration, and modality will begin to highlight the contributing factors to improved function.

### 3.1. Leg Cycling Improves Walking Speed, Endurance, and Balance

**Figure 1:**
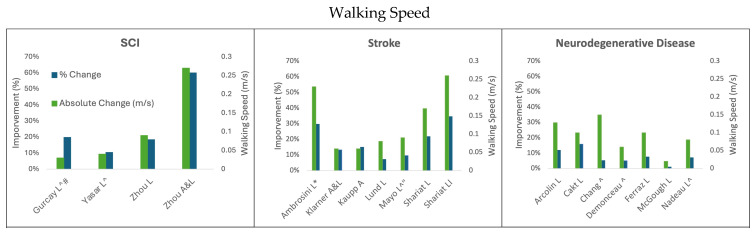
Change in walking speed following cycling interventions in persons with SCI, stroke, and neurodegenerative disease (MS and PD). L = leg cycling; A = arm cycling; A&L = arm and leg cycling; I = interval training; * indicates the median and IQR reported; ^ indicates testing using a self-selected speed; # indicates 20 m test; “ indicates 5 m walk test.

**Figure 2:**
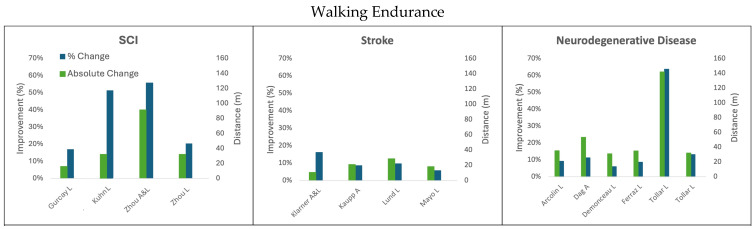
Change in walking endurance following cycling interventions in persons with SCI, stroke, and neurodegenerative disease (MS and PD) using the 6 min walk test. L = leg cycling; A = arm cycling; A&L = arm and leg cycling.

**Figure 3:**
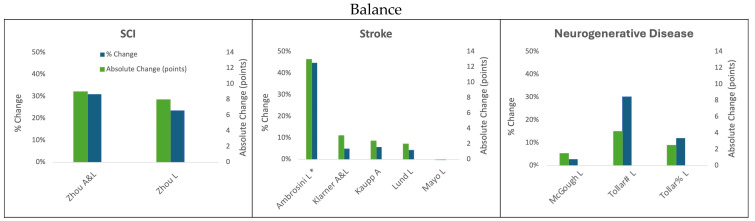
Change in balance following cycling interventions in persons with SCI, stroke, and neurodegenerative disease (MS and PD) using the Berg Balance scale. L = leg cycling; A = arm cycling; A&L = arm and leg cycling; * indicates the median and IQR reported; ; # Tollar et al., 2019 [44]; % Tollar et al., 2020 [45].

The results summarizing the absolute and percent improvement after different cycling paradigms in walking speed, endurance, and balance are discussed below and summarized in Figure 1, Figure 2 and Figure 3, respectively.

#### 3.1.1. Leg Cycling Improves Walking Speed

Increases in walking speed that result from legs-only cycling interventions are similar to those obtained after gait-specific training. This indicates that task-specific interventions are not necessarily required to improve walking function [27,43,64].

Across the studies in Table 1, the dose of leg cycling exercise resulting in clinically significant improvements in walking speed ranged from 336 to 3600 total minutes of cycling training. The factors contributing to this range include the neurological condition, the severity of the condition at the onset of training, and the cycling paradigm (passive cycling, independent cycling, and FES-facilitated cycling). While it is challenging to elucidate the cause of improved outcomes across studies due to the different variables (dose, modality, baseline characteristics of participants, etc.), we provide below a consolidated summary of the findings.

Three studies evaluated the change in walking speed with FES-facilitated leg cycling in persons with incomplete SCI [27,36,46]. Two of these studies [36,46] reported self-selected walking speeds rather than the fastest walking speeds on the 10MWT. These two studies did not show clinically significant changes in walking speed, with improvements of 0.03 m/s (19.8%) and 0.04 m/s (10.5%), respectively. In contrast, the study that tracked the fastest walking speed on the 10MWT [27] demonstrated a clinically significant 0.09 m/s (18.4%) increase in walking speed after the intervention. However, this study had a 12 h higher dose of FES-facilitated leg cycling in one case, and 42 h more cycling in the other [27,50]. Exercise dosage and frequency may be the factors that influenced the greater improvement seen between these studies; thus, future work should track trends in walking speed throughout the intervention period to identify when improvements are achieved relative to the cycling dosage. It may also be important to track the fastest walking speed on the 10MWT following a cycling intervention to identify true improvements in walking speed. Although improvements are noted following leg cycling in SCI, none of these improvements appear to exceed the MCD in walking speed on the 10MWT [51].

Dosage also appears to influence the 10MWT results for people with stroke who engage in legs-only cycling interventions. For example, FES-facilitated leg cycling resulted in a 0.23 m/s (29.9%) improvement based on median (not average) walking speed in participants living with stroke who exercised for 25 min, five times per week, for 3 weeks (375 min of training) [65]. The most similar intervention to this administered 28 min of cycling three times per week for 4 weeks in which 8 min were performed without FES and 20 min were performed with FES-facilitated leg cycling [43]. This intervention reported their results as an average improvement on the 10MWT of 0.17 m/s (22.0%). FES-facilitated leg cycling resulted in much greater improvement than cycling without FES for persons with stroke: even with substantially greater exercise dosages, the studies that did not use FES resulted in less than half the improvement (0.08–0.09 m/s) on the 10MWT test as those that used FES-facilitated leg cycling with lower exercise dosage [29,39,40,43]. This was regardless of whether the 10MWT speed was self-selected or the fastest safe speed [39,40]. In general, leg-cycling interventions for individuals living with stroke required less training time to achieve greater absolute and percent changes in 10MWT than people with SCI when both populations received FES assistance during leg cycling training [27,43].

Improvements in walking speed that result from leg cycling training for participants with PD or MS ranged from 0.02 to 0.15 m/s, or 1.1% to 15.8% [30,31,32,34,35,41,42]. None of these interventions used FES to enhance leg cycling training. For the participants with PD, greater improvements were seen in the studies where the leg cycling intervention was combined with other forms of rehabilitation (see Arcolin et al., 2016 [30] and Ferraz et al., 2018 [35] in Table 1). Regardless of the leg cycling intervention dose and conjunctive therapeutic approaches, no studies achieved the MDC for comfortable walking speed (0.18 m/s) on the 10MWT through the interventional periods [55]. Cycling for 60 min three times a week for 10 weeks resulted in a 1.1% improvement in walking speed (0.02 m/s, n = 38) [41]. In a very intensive program involving 30 min of leg cycling training twice per day in conjunction with an additional 60 min of stretching and strengthening exercise five times per week over 3 weeks, the improvement in walking speed was improved by 0.13 m/s (12.1%) in the participants with PD [30]. Combined physiotherapy and 30 min of leg cycling three times per week for 8 weeks (see Table 1, Ferraz et al., 2018 [35]) improved walking speed on the 10MWT by 0.1 m/s (7.7%). Interval leg cycling composed of 15 sets of 4 min of cycling with 2 min of rest between sets twice per week for 8 weeks similarly demonstrated 0.1 m/s (15.8%) improvement in the 10MWT for participants with MS [31]. It appears that exercise dose, frequency, and intensity all contribute to the amount of improvement in walking speed seen across neurological conditions. FES assistance appears to be a meaningful way to improve the effectiveness of leg cycling.

#### 3.1.2. Leg Cycling Improves Walking Endurance Measured with the 6MWT

Walking endurance is another indicator of improved walking function which can be measured through the 6 min walk test (6MWT). The percent improvement in walking endurance resulting from leg cycling interventions ranged from 6% to 63.7%, and the absolute improvement in distance covered during the test was 16.04–142.00 m across all the reviewed populations [34,36,44].

For persons with chronic incomplete SCI, the study that completed the greatest number of cumulative minutes (3600 completed across 12 weeks) resulted in a 32.12 m (20.4%) increase in walking endurance [27]. In comparison, a study that completed <1/3 of the cumulative training minutes (1080 min over 6 weeks) showed a 16.04 m (17.0%) improvement in walking endurance for individuals with chronic incomplete SCI [36]. In the participants with chronic incomplete SCI who had lower baseline walking endurance, 160 min of FES-facilitated leg cycling resulted in 32.10 m (51.36%) improvement in walking speed [38]. The rate of improvements in walking endurance seen in persons with incomplete SCI because of FES-facilitated leg cycling interventions appears to be relative to the baseline walking endurance of the participants as well as the dosage of exercise [27,36,38]. None of the studies that only engaged the legs in cycling reached the level of improvement of the MCID for individuals with incomplete SCI, which is 36 m [58].

The participants living with stroke had a 28.61 m (9.6%) increase in walking endurance after completing a leg cycling intervention administered for 36 min a day three times per week for 12 weeks [39]. An 18.30 m (5.7%) improvement was also seen with 15–30 min of daily training for one year [40]. Very few studies on leg cycling interventions in participants with stroke conducted the 6MWT, and those that did collect the measurement did not show results that surpassed the threshold of the MCID [39,40,59].

The persons with PD experienced the largest absolute and percent increase in walking endurance after the leg cycling training across the reviewed populations and surpassed the 82 m MCID [44]. The successful intervention reporting this outcome used 60 min of leg cycling five times per week for 5 weeks (1500 min), resulting in a 142 m (63.7%) increase in walking endurance [44]. However, three other leg cycling interventions studied in the participants with PD showed 6%, 8.6%, and 9.2% improvement in the 6MWT where all three studies had absolute changes in distances between 31.0 and 35.2 m [30,34,35]. These less effective studies had shorter training periods (<45 min) relative to the successful intervention. Surprisingly, among these three studies, the one that demonstrated the lowest absolute and percent improvement in walking endurance (31 m, 6%) had the greatest cycling exercise dosage (1260 min) [34]. However, the two other studies [30,35] used physical therapy in conjunction with cycling paradigms (900 min and 720 min) to obtain similar absolute improvements in shorter time-frames.

Only one study tracked the 6MWT in participants with MS and the leg cycling group improved by 32.1 m (13.1%) with 5× 1 h sessions per week for 5 weeks [45]. This improvement is within the range of reported MCIDs for persons with MS (21.56–55.06 m) and may be clinically significant [57,60]. Notably, this intervention was conducted by the same first author as the only group to show clinically significant results (per the MCID) in participants with PD on the 6MWT [44,45]. Improvements in walking speed appear to also be linked to the overall dose of exercise across clinical conditions.

#### 3.1.3. Leg Cycling Improves Balance on the BBS

Balance, commonly assessed using the BBS, is important to both walking function and reduced fall risk, and can improve in clinical populations through longitudinal engagement with cycling training.

Individuals with SCI improved their balance by eight points (23.5%) during a leg cycling intervention of 60 min of training five times a week for 12 weeks [27]. There is no MDC or MCID specific to SCI for the BBS; however, eight points exceed the MDC and MCID for all the other clinical groups, so it is reasonable to hypothesize that this change is clinically significant.

In one out of three studies that used leg cycling training paradigms in individuals living with stroke, clinically significant results were found [29]. Specifically, training 25 min five times a week for 3 weeks resulted in a 13-point (44.8%) increase in the median BBS score [29]. This improvement exceeds the expected threshold of the MCID for persons with acute stroke by five points [41]. This was the only study among those reviewed that followed persons during the acute stroke phase, and also the only study that reported BBS by comparing the median scores instead of the average scores on the assessment. Two studies that investigated leg cycling paradigms in chronic stroke did not result in improvements in BBS scores [39,40]. In the first of these two reviewed studies, training 26 min three times per week for 12 weeks resulted in an average increase of two points on the BBS from a sample of participants with baseline BBS scores ranging from 14 to 51 points [39]. No significant change in the BBS was seen through 15–30 min of daily training for one year by individuals living with chronic stroke; however, the initial BBS scores of participants in this study were near neurologically intact values (51.4 ± 5.1 points), leaving very little room for improvement [40]. Moreover, the study reports that 23.3% of the individuals in the leg cycling cohort reached the MCID threshold [40].

The people with PD obtained a 1.5 average point increase on the BBS as a result of a 10-week intervention with 60 min of training three times per week, which is below the MDC of 5 points for the population [41]. Balance also improved both for the individuals living with PD and MS when they completed 60 min of leg cycling five times a week for 5 weeks [44,45]. For the participants with PD, this intervention resulted in an average 4.2 point improvement which is below the MDC for the population [44]. Similarly, the improvement seen in persons with MS was just shy of the MCID for the population (3 points) with a 2.5 average point increase emerging from the intervention [45].

Overall, leg cycling has beneficial effects on the clinically important walking parameters of speed, endurance, and balance across people with neurological trauma or disease. While these improvements are impressive, greater improvements in walking function can be attained when the arms are actively and simultaneously engaged with the legs in cycling training, which is the topic of the next section.

### 3.2. The Addition of the Arms During Cycling Improves Functional Walking, Balance, and Electrophysiological Outcomes Relative to Legs-Only Cycling

One critical consideration when employing a cycling intervention is the inclusion of the arms as part of the training. Arm movements are inherent to normal walking and as a result, are vital when administering rehabilitative interventions for the improvement of walking capacity. The combination of A&L movement affords the opportunity to enhance interlimb coordination and provides meaningful changes that can build upon the benefits that would arise from solely performing leg cycling. Evidence suggests that the basic neural elements controlling and coupling the arms and legs during coordinated rhythmic movements are similar to those in quadrupedal animals, and engaging the arms to enhance proprio-spinal circuitry may be beneficial in rehabilitation [6]. These benefits have been documented in interventional studies that are related to recovery after SCI and stroke [27,66].

A&L cycling has been directly compared to legs-only cycling in a study in participants with SCI where training was for 60 min a day five times a week for 12 weeks. One group in this study received an FES-facilitated legs-only intervention, while the other group simultaneously cycled with their arms during FES-facilitated leg cycling. While legs-only cycling using the same training schedule resulted in a 0.09 m/s (18.4%) improvement in the 10MWT, the inclusion of the arms resulted in a 0.27 m/s (60%) improvement in walking speed [27]. Similarly, while performance on the 6MWT increased by 32.12 m (20.4%) in the legs-only cycling group, A&L cycling increased endurance by 91.58 m (55.7%) [27]. Critically, the group that completed the intervention that included the arms surpassed the MCID for walking endurance, but the legs-only cycling group did not. Moreover, balance improved by eight points on the BBS (23%) in the legs-only cycling group compared to a nine-point (31%) improvement in the A&L cycling group [27]. Nonetheless, it is unclear whether a single point difference in the BBS indicates a substantial change in balance for persons with incomplete SCI.

In stroke, the participants who exercised with A&L cycling 30 min a day, 3 days a week for 5 weeks saw a 0.06 m/s 13.3% improvement in their 10MWT speed [66]. This intervention did not include FES facilitation, which has been shown to improve walking outcomes substantially in leg cycling interventions for persons with stroke [29,39,40,43,66]. The most closely matched leg cycling study in the literature involving participants with stroke was a paradigm consisting of 28 min a day of cycling, three times per week for 12 weeks [43]. The leg cycling intervention showed a 0.08 m/s (22%) increase in the 10MWT compared to the A&L cycling protocol, although this slight difference in improvement in walking speed may be due to the difference in the duration of the training paradigms. This same study demonstrated the lowest absolute change on 6MWT of any study in stroke using legs-only cycling or arms-only cycling, but the percent change was the highest of any of these studies (10.68 m, 16.1%) [37,39,40,66]. Future investigations should demonstrate A&L cycling in persons with stroke against a leg cycling cohort of participants that are well matched with their baseline characteristics. A&L cycling in the participants with stroke also produced the greatest absolute change in the BBS score (3.1 points) in the non-FES-facilitated interventions [37,39,40,66].

A&L cycling also demonstrates improvements in stretch reflex modulation in persons with stroke. Before training, stretch reflex modulation was low in the tibialis anterior muscle but the modulation increased after training [28]. Improved modulation of the tibialis anterior is important for reducing foot drop that can lead to trips and falls while walking, and tight regulation between supra-spinal centers and this muscle is necessary for ongoing corrective control [43].

The improvements attained by legs-only and A&L cycling training following SCI highlight that cycling training translates to improvements in walking, and that the simultaneous inclusion of the arms in the training is a key factor for further enhancing walking speed, endurance, and balance after neural injury [27]. Considering that legs-only cycling has benefitted persons with PD and MS [30,31,35], it is worth investigating how the incorporation of the arms could further benefit these clinical groups. Additionally, an A&L cycling investigation should be conducted when FES is employed in the legs of persons with stroke [43,66]. Investigations directly comparing paradigms with legs-only and A&L cycling would provide clarity into the rehabilitative differences that emerge when the arms are included in the training. The identification of the most appropriate dose of training should also be considered in future research investigations.

### 3.3. Enhanced Interlimb Coupling After Arm and Leg Cycling

Interlimb coupling governs the coordination between the arms and legs during movement. This bidirectional coupling between lumbar and cervical spinal locomotor centers is vital for coordinated locomotion [6]. This coupling can be measured by examining how activity in the arms/legs modulates evoked response in the legs/arms [67,68,69]. Studies on the modulation of motor evoked potentials or spinal reflexes in humans may shed some light on how arm/leg proprio-spinal interlimb coupling affects functional changes following a rehabilitation intervention [16,28,70,71,72].

In people with an intact nervous system, there is a general enhancement of excitability in the cortico-spinal tract to the arms/legs with rhythmic movement of the opposite limbs occurring [72] and a general suppression in spinal reflexes [12,67,73,74,75,76]. In persons with incomplete SCI, A&L cycling training increased cortico-spinal tract excitability to the tibialis anterior muscle more so than legs-only cycling [72], reducing the potential impact of foot drop on walking function.

The modulation of interlimb spinal reflexes suggests the presence of intersegmental linkages between the cervical and lumbar spinal cord regions through proprio-spinal connections [4,5,6,11,77,78]. After A&L cycling training in persons with stroke, increased suppression of cutaneous reflex pathways in the soleus, posterior deltoid, and tibialis anterior muscles of the more affected limbs was evident [28,66]. In fact, before training, no suppression (modulation) of cutaneous reflex activity was present at all, revealing a revitalization of the normal suppression seen in neurologically intact individuals in this reflex pathway after A&L cycling training. In addition to cutaneous reflexes, the H-reflex, which is the electrical analog of the stretch reflex, is also suppressed after both legs-only and A&L cycling training in persons with incomplete SCI [16]. These changes in interlimb coupling accompanied by improved functional outcomes with A&L cycling highlight the utility and importance of engaging the arms in a locomotor rehabilitation paradigm.

### 3.4. Arm Cycling Alone Can Improve Walking and Enhance Interlimb Coupling

Improvements produced by the incorporation of the arms into cycling paradigms have demonstrated improved outcomes for persons with SCI relative to leg-specific interventions, begging the question of what the arms in isolation can do to modulate leg movements to obtain the functional restoration of walking.

Within rehabilitation cycling interventions, arms-only cycling has demonstrated a variety of benefits that can improve the rehabilitation of walking in individuals experiencing stroke [37] and in individuals with PD [33]. For example, an arms-only cycling training program for participants with stroke where the participants exercised for 30 min three times per week for 5 weeks resulted in significant improvements in the 10MWT (Figure 1), the 6MWT (Figure 2), and the BBS (Figure 3). The time it took to walk 10 m decreased from 24.5 s to 20.8 s, which was an improvement of 0.06 m/s (15.1%) [37]. In the 6MWT, walking distance increased from an average of 245.1 m to an average of 266.1 m (a 21 m, 8.5% increase) [37]. There was also a 5.7% improvement in the BBS with the participants improving an average of 2.4 points (score of 41.5 to 43.9) [37]. In persons with PD, arm crank ergometer training for 8 weeks resulted in an increase in walking distance of 53.31 m, an improvement of 11.2% [33]. This improvement on the 6MWT is notably 18.11 m–22.31 m greater than 75% of the legs-only cycling studies in persons with PD [30,34,35]; however, the improvements seen through arms-only cycling are still 29 m below the MDC for the 6MWT in persons with PD. Cycling with the arms only also improved participant performance on the Timed-Up-and-Go test by 1.74 s (17.5%) [33]. The participants who arm-cycled also obtained significant improvements in their motor impairment using the Unified Parkinson’s Disease Rating Scale, VO_2_ max, Beck Depression Index, Falls Efficiency Scale, and quality of life using the Parkinson’s Disease Questionaire-39 [33].

The improvements in walking were also exhibited through a normalization of reflex modulation in the participants with stroke, where there was increased suppression in the tibialis anterior pathways on both the less- and more-affected sides after training [37]. Before training, no suppression (modulation) of cutaneous reflex activity was present, but the modulation became evident after training, revealing a revitalization of the normal suppression found in this reflex pathway. Improved modulation in the stretch reflex pathway was also seen with arm cycling training revealing enhanced interlimb coupling [37]. After arm cycling training interventions, there was a greater intersegmental suppression of soleus stretch reflexes from arm cycling [37] which is closer to what is found in neurologically intact populations [12,67,73,74,75].

Arms-only cycling training activates interlimb networks that contribute to the coordination and improvement of rhythmic walking. This increased coordination in rhythmic walking highlights the importance of training interlimb connections and demonstrates the improvements that arms-only cycling can have on overground walking function and balance after stroke or PD [33,37]. These studies have demonstrated clear evidence of the important contributions of the arms to inputs onto lumbar circuitry that can induce long-term changes in functional performance. Further investigations are needed to identify the effects of arms-only and legs-only cycling as well as the additive effects of A&L cycling in people with PD and MS.

## 4. Methods to Further Enhance Proprio-Spinal Connectivity to Improve Walking After Neural Injury

### 4.1. Functional Electrical Stimulation to Enhance the Effects of Arm and Leg Cycling

Regular physical activity is a necessity for those living with SCI as they face secondary complications including hypertension, diabetes, muscle atrophy, and bone density loss. The ability to perform exercise is directly linked to the severity and level of the injury and is restricted by cardiac elements [79,80,81,82,83,84]. However, regular physical activity through activities such as cycling or rowing engages muscles across the body and leads to improved physical fitness [85]. For those who lack motor drive in the leg muscles, adding FES to the primary hip and knee muscles can produce functional cycling movements [86]. Considerable evidence has shown the beneficial effects of FES-facilitated cycling where specific muscles are targeted with direct electrical stimulation. The electrical stimulation of 140 mA and frequency range from 33.3 to 50 Hz led to improvements in body composition after 12 months of FES-facilitated cycling [87]. Thirteen men with complete SCI participated in a three-phase program with stimulating electrodes placed over the quadriceps, hamstrings, and gluteal muscles to assess changes in muscle mass. Computed tomography of the legs revealed an increase in muscle mass and muscle cross-sectional area [79]. Persistent FES-facilitated leg cycling training contributes to a healthier life, reduces secondary risks, and induces positive changes in heart rate, stabilization of blood pressure, and improvements in blood pressure [80], thus combating major secondary concerns of muscle paralysis and cardiorespiratory dysfunction. FES-facilitated cycling may also improve both sensory and motor function, and may reduce spasticity [81].

### 4.2. Implementation of Cycling Assisted by Functional Electrical Stimulation in an Acute Care Setting

The beneficial effects of leg cycling could be enhanced if a rehabilitation protocol is implemented early after neural injury. Physical inactivity, bed rest, and prolonged immobilization after injury can have significant consequences on the musculoskeletal system, as muscle wasting occurs within the first week of hospitalization [82]. Adaptive neuroplasticity begins immediately after injury and is highly sensitive to repetitive motor inputs [83,84]. By minimizing the time between injury and intervention, practitioners can maximize positive neuroplasticity and improve functional outcomes [83,84]. Although the most appropriate method for acute rehabilitation has not been determined, recent work has explored the use of in-bed cycling for acute neural injuries. Conducting cycling in a supine position reduces safety concerns and simplifies motor relearning and training for patients [84]. In-bed cycling can be conducted with the use of FES and motor support to produce sufficient muscle contraction necessary for cycling [88]. Although the most effective progression and parameters for FES are still being explored, an individualized approach is strongly recommended [88]. Rhythmic movement of the limbs in the acute phase was shown to be feasible and could potentially influence neurofunctional outcomes, quality of life, length of stay, and reduce secondary complications [84,88]. Ultimately, more robust research is needed to determine the most appropriate methods for acute leg cycling and the benefits of FES-facilitated cycling. Acute-setting leg cycling or arm cycling could address several barriers to patient care and has the potential to elicit positive responses in recovery and patient outcomes.

### 4.3. High-Intensity Interval Training to Enhance Improvements of Arm and Leg Cycling

High-intensity interval training (HIIT) was incorporated into FES-facilitated legs-only cycling in study participants with stroke and resulted in larger improvements in functional walking outcomes than a linear pattern of training [43]. The training was administered for 28 min, three times per week for 4 weeks with HIIT as a variable.

HIIT improved walking speed by 12.7% (0.09 m/s) over linear training and Timed Up and Go (TUG) by 28.2%. The linear training resulted in a 0.17 m/s improvement on the 10MWT compared to a 0.26 m/s improvement in the HITT group [43]. This is the second largest absolute change in walking speed obtained through any of the reviewed cycling interventions, second only to the FES-facilitated A&L cycling in persons with SCI (0.27 m/s increase) [27,43]. Walking endurance was not measured in the study and balance, measured through the single-leg stance test, had no difference between the HIIT and linear training groups. The absolute difference in the 10MWT results between the HIIT and linear training groups was 0.09 m/s, which in itself is greater than the MCID for persons with chronic stroke [43,50]. Interval leg cycling with a format of three sets of 12 min at 75% heart rate reserve intensity with 5–10 min rest breaks in between (936 total minutes) increased walking speed by 0.08 m/s (7.4%) for individuals living with stroke [39].

In a study with participants with PD, HITT was incorporated into a legs-only cycling intervention where the paradigm involved at least one 30–45 min of cycling at 50–60% of peak workload (continuous session) per week and at least one interval cycling training session 16–24 min long plus warm up and cool down at 40–50% peak workload for 10 min each [34]. The results did not show an enhancement with HITT for the participants with PD beyond what the other interventions were able to accomplish without interval training [30,32,34,35]. In contrast with the study that used HITT to improve a cycling paradigm following stroke, this study did not use FES, so the outcomes associated with the HITT training may be the result of improving responses to FES by performing short stints with FES and allowing recovery afterward [34,43].

Studies with MS do not make direct comparisons in the functional outcomes of walking from interventions that do and do not incorporate HITT. Two studies of people with MS performing leg cycling were reviewed: one study used HITT and reported walking speed [31] and the other conducted linear training and reported balance and walking endurance [45]. The study using HITT was structured as 15 sets of 4 min of cycling where 2/4 were high intensity and 2/4 were low resistance or rest minutes. This paradigm created the greatest percent improvement (15.8%) on the 10MWT and the third highest absolute improvement (0.1 m/s) amongst studies involving participants with motor-system neurodegenerative conditions [30,31,32].

It is unclear how a combination of HIIT and FES could impact walking outcomes in people experiencing SCI, PD, or MS. HIIT with and without FES facilitation of legs-only cycling should be studied across various populations. Additionally, these two potential cycling enhancement tools should be investigated within the context of A&L cycling and arm cycling, and in conjunction with other adjunct therapies to determine the most effective intervention strategies.

### 4.4. Paired Spinal Cord Stimulation to Improve Walking After Neural Injury

#### 4.4.1. Epidural Spinal Cord Stimulation (eSCS)

Epidural spinal cord stimulation (eSCS), while an invasive and expensive approach, has shown dramatic improvements in walking rehabilitation when paired with exercise rehabilitation strategies. eSCS is a form of neuromodulation that is generally applied within the epidural space of the spinal cord on the dorsum of the dura mater. Typically, eSCS is either placed via cylindrical linear multicontact electrodes that are implanted percutaneously or through multicolumn paddle arrays that are implanted via laminectomy [89]. Once implanted, eSCS may provide the ability to restore functional and volitional lower extremity movements after SCI by indirectly activating motor neurons, even in the case of chronic complete paralysis. The use of eSCS has been shown to modulate neuronal circuits in persons with motor-complete SCI including cortico-spinal [90,91,92], proprio-spinal [93,94], and cortico-reticulo-spinal [95] tracts. The resulting neuroplasticity is thought to improve spinal motor output and volitional movements even in cases of severely reduced supra-spinal input, without negatively impacting residual motor function [96,97,98,99,100,101].

An early case study demonstrated that eSCS can facilitate locomotor recovery for an individual with a C5–C6 chronic incomplete SCI [102]. The training program initially focused on partial body weight-supported treadmill training and progressed to overground training with a front-wheeled walker as the participant’s ability to support weight and generate coordinated stepping movements improved. Therapy was provided for up to 2 h per day, 5 days per week for 12 weeks. There was progressive improvement in stepping patterns over the initial 12 weeks of training as demonstrated by significant bilateral improvements in step length, cycle time, swing time, and stance time [103]. These improvements allowed treadmill training speed to be increased up to 0.68 m/s while static partial weight support levels were reduced to less than 20% of body weight.

More recently, eSCS applied to the lumbar spinal cord, in conjunction with intensive locomotor training, allowed persons with clinically motor complete SCI to step over ground for short distances [97,104,105]. This demonstrated that dormant neurons that survive the injury may be reengaged with spinal neuromodulation, and produce stepping-like movements [103,106]. Consequently, the combination of eSCS with rehabilitative exercise paradigms offers tremendous promise.

#### 4.4.2. Non-Invasive Transcutaneous Spinal Cord Stimulation (tSCS)

Transcutaneous spinal cord stimulation (tSCS) is a non-invasive approach aimed at facilitating previously inaccessible spinal circuitry with electrical stimulation applied over the spinal cord [107]. An increasing body of evidence indicates that tSCS paired with an exercise modality can enhance recovery after neurotrauma. In case studies and small clinical trials, tSCS has produced locomotor-like stepping movements [108,109], and improved walking function [110,111,112] in participants with neurological deficits including incomplete and complete SCI, stroke, and cerebral palsy. Building on these changes seen within a single session of stimulation, a recent case study highlighted that two months of cervical and lumbar tSCS combined with intensive training improved walking after chronic incomplete SCI compared to locomotor training alone. The intensive locomotor training included overground walking and a treadmill training program. The improvement in the 6MWT distance was 3× greater with the addition of tSCS compared to training alone (Participant 1: +37% of baseline during training only vs. +124% of baseline during stimulation + training; Participant 2: +24% of baseline vs. +65% of baseline), while balance and step length improved similarly [69]. After intensive training, the participants required less assistance while walking, had better balance, and improved their stair management.

In children with cerebral palsy, locomotor training was completed in both a control group (treadmill training only) and in the experimental group that received simultaneous tSCS over T11 and L1 spinous processes during locomotor treadmill training [110]. After spinal cord stimulation in the experimental group only, there was an incremental increase in knee torque while the amplitude of hip motion increased in both groups. A decrease in the co-activation of hip muscles and distal muscles of the lower extremities was observed in the experimental group, while in the control group, co-activation decreased only in the hip muscles [113]. The results support the idea that locomotor function can be improved significantly with the combination of training and tSCS than with training alone.

Furthering this evidence, a recent case series assessed 10 individuals with chronic (>1 year) motor incomplete SCI who completed 23 sessions of 2 h therapy over 8 weeks with tSCS applied for 30 min [112]. Importantly, the combined intervention was feasible in an outpatient clinical setting and all the participants tolerated the tSCS with minimal adverse events. Changes in walking speed, as measured by the 10MWT (0.56 ± 0.29 m/s to 0.72 ± 0.36 m/s; 28.6% increase), and changes in walking endurance, as measured by the 6MWT (149.88 ± 99.87 m to 194.53 ± 106.56 m; 30.2% increases), improved, demonstrating both a statistically and clinically significant change [112]. This is an important step to highlight the feasibility and potential effectiveness of tSCS use as an adjunct walking-based therapy.

The potential for improving mobility outcomes through cervicolumbar connectivity by combining A&L cycling with tSCS remains a key avenue for future research. Instead of treadmill walking training, a combination of tSCS and A&L cycling training may be able to improve interlimb connections after neural injury. However, studies that directly compare an exercise intervention with tSCS and exercise with larger sample sizes are vital in determining the potential scale of these improvements.

#### 4.4.3. Combining Invasive and Non-Invasive Spinal Stimulation

Excitingly, although still in the early stages of testing, combined cervical tSCS with lumbosacral eSCS was shown to influence both spinal excitability and interlimb coupling within a single session of stimulation [113]. These neurophysiological alterations were accompanied by an improved range of motion, amplitude of motor output, and coordination during non-weight bearing and weight bearing intentional stepping for individuals with motor complete SCI.

### 4.5. Development of Coordinated Arm and Leg Rehabilitation Robotics

Most currently available rehabilitation robots provide therapy only for the lower limbs or for the upper limbs independently. However, recent research has revealed the advantages of cable-driven robotic devices for all four limbs. Due to characteristics such as low inertia, easy configuration, and high speed and power output, belt actuation is applied in robotic arm rehabilitation [114,115] and gait restoration [116,117]. Soft interfaces from the cables with the user remove the motion constraints brought by conventional rigid exoskeletons. Belt actuation can also produce natural compliance in movement control [118]. Therefore, belt actuation can potentially provide a comfortable and compliant human–robot interaction. One company, Healing Innovations, has developed a robotic-assisted gait training device that synchronizes upper-body and lower-body movement. The device has seated and standing capabilities and body weight support, and can be used in both inpatient and outpatient settings (Healing Innovations, n.d.), providing the opportunity for early training after injury [119,120,121]. Much more work is still needed to support early rehabilitation of arm/leg movement in acute care settings.

Recently, a group explored the use of kinematic analysis, mechanical design, control development, and experimental evaluation of two test rigs for the generation of various stepping movements in a supine-lying position and walking-like stepping in a side-lying position [84,121]. Using innovative mechanical configuration and automatic control systems, various stepping movements were produced in supine-lying and side-lying positions, demonstrating technical feasibility. Future work will focus on kinetic analysis of the system and closed-loop position control to compensate for belt flexibility and friction. The implementation of various leg movements will be followed by the development of arm movement. The automatic control system and mechanical configuration of the two test rigs will be extended and applied to the development of the overall robotic rehabilitation platform. This avenue of exploration provides an exciting opportunity for synergistic improvements in function after neural injury or disease including MS, PD, SCI, and stroke.

## 5. Conclusions and Future Directions

The inclusion of the arms in rehabilitation paradigms for gait retraining is vital to maximize the functional improvements after neural injury or disease. While arms-only or legs-only cycling can provide clinically meaningful improvements in walking speed in some cases for persons with SCI or stroke, the combination of arm cycling with FES-facilitated leg cycling in synchrony enhances improvements in walking speed, distance, and functional balance in people with SCI beyond what has been shown in rehabilitation paradigms focusing on the legs only. While the currently available results provide exciting proof of principle, large multi-center trials are required to ensure this rehabilitation approach is effective, leading to its uptake into rehabilitation practice. Furthermore, an improved understanding of the impact of time since injury and the level of the lesion after SCI are factors that require further investigation. Integrating simultaneous A&L cycling training in acute care settings may enhance the rate and magnitude of neurorecovery. Moreover, combining A&L cycling with tSCS or eSCS may further improve functional outcomes. Implementing HIIT in A&L cycling would likely enhance both cardiac and neurorecovery, and integrating simultaneous A&L functionality in rehabilitation robotics may enhance functional improvements and neural connectivity. To date, the effect of A&L cycling on the core and stabilizing muscles of the trunk has been overlooked and requires further investigation. The use of each paradigm depends on the clinical population, the availability of equipment and expertise, the associated cost, and the potential mechanism of action to facilitate enhanced recovery. Furthering our understanding of how these interventions alter interlimb connectivity is vital for their deployment in a purposeful and targeted manner. Considering factors such as the most effective time for implementation, the intensity of the training activity, and technologies that can be paired with a chosen rehabilitation paradigm will allow for improved outcomes in the most efficient manner.

## Figures and Tables

**Table 1:** Summary of included studies organized alphabetically by author’s last name.

Author Name [Reference #]	Number of Participants	Clinical Group	Training Mode	Training Frequency	Type of Cycling
Ambrosini et al., 2020 [29]	9	Stroke	25 min sessions	5× per week for 3 weeks	FES Leg Cycling
Arcolin et al., 2016 [30]	16	Parkinson’s Disease (PD)	30 min twice per day and 60 min of stretching, strengthening, and balance exercises	5× per week for 3 weeks	Leg Cycling
Cakt et al., 2010 [31]	14	Multiple Sclerosis (MS)	15 sets of 4 min of cycling (2 min on high resistance followed by 2 min on low resistance or 2 min of rest)	2× per week (non-consecutive days for 8 weeks	Leg Cycling
Chang et al., 2018 [32]	13	PD	Session 1 = 15 min Session 2 = 20 min Session 3 = 25 min Session 4–8 = 30 min Session 9–12 = 35 min Session 13–16 = 40 min	2× per week for 8 weeks	Leg Cycling
Dağ et al., 2021 [33]	13	PD	60 min	3× per week for 8 weeks	Arm Cycling
Demonceau et al., 2017 [34]	16	PD	At least one 30–45 min of cycling at 50–60% of their peak workload (PWL)(continuous session) per week AND At least one interval cycling training session 16–24 min long plus warm up and cool down at 40–50% peak workload for 10 min each	2–3× per week for 12 weeks with at least one rest day between sessions	Leg Cycling
Ferraz et al., 2018 [35]	20	PD	50 min physiotherapy sessions with 30 min of leg cycling, 10 min of stretching, 5 min of calisthenics, and 5 min of respiration exercises	3× per week for 8 weeks	Leg Cycling
Gurcay et al., 2022 [36]	15	Chronic spinal cord injury (SCI)	30 min sessions with 20 min of cycling, a warm up (5 min), and a cool down (5 min)	3× per week for 6 weeks	FES leg cycling
Kaupp et al., 2018 [37]	19	Stroke	30 min	3× per week for 5 weeks	Arm Cycling
Klarner et al., 2016 [28]	19	Stroke	30 min	3× per week for 5 weeks	Arm and Leg Cycling
Kuhn et al., 2014 [38]	5	Incomplete SCI (AIS C/D)	20 min	2× per week for 4 weeks	FES Leg Cycling
Lund et al., 2018 [39]	13	Stroke	3 sets of 12 min at 75% heart rate reserve intensity with 5–10 min rest breaks in between	3× per week for 12 weeks	Leg Cycling
Mayo et al., 2013 [40]	28	Stroke	Starting at a minimum of 15 min and building up to 30 min of training. Advised to exercise on the 6–20 scale Borg scale at an 11–15 intensity.	Daily for one year	Leg Cycling
McGough et al., 2016 [41]	38	PD	60 min	3× per week for 10 weeks	Leg Cycling
Nadeau et al., 2017 [42]	19	PD	Initially at 20 min at 60% exertion building to 40 min at 80% exertion over the 12 weeks	3× per week for 12 weeks	Leg Cycling
Shariat et al., 2021 [43] Linear	14	Stroke	28 min (8 without FES stimulation and 20 with stim) continuously	3× per week for 4 weeks	FES Leg Cycling
Shariat et al., 2021 [43] Interval	16	Stroke	28 min (8 without FES stimulation and 20 with stim) 20 min with stimulation are split into 4 × 5 min intervals	3× per week for 4 weeks	FES Leg Cycling
Tollár et al., 2019 [44]	25	PD	60 min including 5 min warm up and 5 min cool down	5× per week for 5 weeks	Leg Cycling
Tollár et al., 2020 [45]	14	MS	60 min	5× per week for 5 weeks	Leg Cycling
Yaşar et al., 2015 [46]	15	Incomplete SCI (AIS C/D)	60 min	3× per week for 16 weeks	FES Leg Cycling
Zhou et al., 2018 [27] Arm and leg cycling	7	Incomplete SCI (AIS C/D)	60 min	5× per week for 12 weeks	Arm and FES Leg Cycling
Zhou et al., 2018 [27] Legs-only cycling	8	Incomplete SCI (AIS C/D)	60 min	5× per week for 12 weeks	FES Leg Cycling

## Data Availability

All the data described in this review were obtained from peer-reviewed publications available in PubMed.
